# KATANIN 1 Is Essential for Embryogenesis and Seed Formation in Arabidopsis

**DOI:** 10.3389/fpls.2017.00728

**Published:** 2017-05-05

**Authors:** Ivan Luptovčiak, Despina Samakovli, George Komis, Jozef Šamaj

**Affiliations:** Centre of the Region Haná for Biotechnological and Agricultural Research, Faculty of Science, Palacký University OlomoucOlomouc, Czechia

**Keywords:** Arabidopsis, development, katanin, embryo, embryogeneis, seed, microtubule

## Abstract

Cytoskeletal remodeling has a fundamental role, especially during transitional developmental stages when cells rapidly adopt new forms and roles, like gametogenesis, fertilization and concomitant embryogenesis and seed formation. KATANIN 1, a microtubule severing protein, fulfills a major regulatory mechanism of dynamic microtubule turnover in eukaryotes. Herein, we show that three well-established *KATANIN 1* mutants, *fra2, lue1* and *ktn1-2* collectively display lower fertility and seed set in Arabidopsis. These lower fertility and seed set rates of *fra2, lue1* and *ktn1-2* mutants were correlated to abnormalities in the development of embryo proper and seed. Such phenotypes were rescued by transformation of mutants with functional *pKTN1::GFP:KTN1* construct. This study significantly expands the already broad functional repertoire of KATANIN 1 and unravels its new role in embryo and seed development. Thus, KATANIN 1 significantly contributes to the fertility and proper embryo and seed formation in Arabidopsis.

## Introduction

In angiosperms, seeds arise from a fertilized ovule and consist of the following parts: the embryo which arises from the zygote, the endosperm which arises from the fertilization of the diploid central cell by haploid sperm cell and the integuments. Cell division pattern pinpoints the development and growth of all three parts and for this reason is crucial for both embryonic and post-embryonic plant development (Jürgens and Mayer, 1994; ten Hove et al., 2015).

The regulation of microtubule dynamics is essential for numerous cellular functions. The ability to accurately control microtubule number, the rate of microtubule assembly and disassembly, and the assembly of microtubule networks governs cellular processes including differentiation, division and migration. A major regulatory mechanism of dynamic microtubule turnover in eukaryotes, involves microtubule severing by means of AAA-ATPase family proteins including katanin (McNally and Vale, 1993; Burk et al., 2001), fidgetin (Mukherjee et al., 2012) and spastin (Roll-Mecak and Vale, 2005). In plants as represented by the genetically tractable model *Arabidopsis thaliana*, only katanin has been identified as a microtubule severing protein (Burk et al., 2001; McClinton et al., 2001), and was shown thereon to play central roles in mechanisms governing microtubule organization. Typically, the mammalian AAA-ATPase katanin is assembled by a catalytic subunit of roughly 60 kDa (p60) and a structural 80 kDa subunit (p80; Hartman et al., 1998). For severing activity, katanin forms hexameric rings on the surface of microtubules and exerts its catalytic activity using ATP hydrolysis (Hartman and Vale, 1999). In the Arabidopsis genome, only the p60 subunit is expressed (designated as KATANIN 1; although four putative p80 orthologs have been identified; Keech et al., 2010) and it is capable of severing microtubules in an ATP-dependent manner (Stoppin-Mellet et al., 2007). At the cellular level, the severing activity of KATANIN 1 was shown to regulate important aspects of plant microtubule organization (Nakamura, 2015). KATANIN 1 was shown to sever nascent, γ-tubulin-nucleated microtubules, growing from the walls of pre-existing microtubules (Nakamura et al., 2010) and also microtubules that are crossing each other during their dynamic excursions (Wightman and Turner, 2007; Soga et al., 2010; Lindeboom et al., 2013; Zhang et al., 2013) favoring in this way the biased parallel arrangement of cortical microtubules. Moreover, KATANIN 1 activity favors microtubule bundle formation (Stoppin-Mellet et al., 2006) and can be modulated by other microtubule binding proteins like SPIRAL2 (Wightman et al., 2013).

Previous studies have revealed that Arabidopsis *KATANIN 1* mutants display pleiotropic phenotypes with defects affecting almost all vegetative organs (Bichet et al., 2001; Burk et al., 2001; Burk and Ye, 2002; Webb et al., 2002; Bouquin et al., 2003; Panteris et al., 2011; Panteris and Adamakis, 2012; Lindeboom et al., 2013; Zhang et al., 2013; Abu-Abied et al., 2015a,b). However, the role of KATANIN 1 in Arabidopsis embryogenesis and seed set regulation has not been addressed so far. To gain insight to KATANIN 1 role we analyzed defects in embryogenesis and seed formation in well established *fra2, lue1* and *ktn1-2* mutants. Mutants *fra2* and *lue1* are rather similar as in both a truncated p60 is produced: in *fra2* due to a deletion at nucleotide 2329 (Burk and Ye, 2002) and in *lue1* because of a single base change producing a non-sense mutation at amino acid 394 (Bouquin et al., 2003). Mutant *ktn1-2* contains a single T-DNA insertion after the 147th nucleotide in the 5th exon of *KATANIN 1* (Nakamura et al., 2010). In this study, we quantitatively surveyed several developmental aspects including embryogenesis and seed formation in above mentioned *KATANIN 1* mutants. Phenotypical abnormalities in embryogenesis and seed formation of *KATANIN 1* mutants were rescued by complementation of these mutants with *pKTN1::GFP:KTN1* construct. Obtained results strongly supported a new role of KATANIN 1 in the embryogenesis and proper seed formation in Arabidopsis.

## Materials and Methods

### Plant Material

*Arabidopsis thaliana* wild type Columbia (Col-0) ecotype, *fra2, lue1* and *ktn1-2*, T-DNA mutant were used. For germination, Col-0 and mutant seeds were surface sterilized, plated on 0.8% w/v Phytagel^®^ solidified ½12 Murashige and Skoog medium (½12 MS) with 1% w/v sucrose, stratified for 1–4 days at 4°C and subsequently transferred to environmental chamber with controlled light/dark cycle, temperature and humidity.

### Pollen Viability Analysis with Alexander Solution Staining

For analysis of pollen viability, stamens from floral buds were placed on a microscopic slide. A few drops of Alexander stain buffer (95% v/v ethanol, 10 mL; Malachite green (1% w/v in 95% v/v ethanol), 1 mL; Fuchsin acid (1% w/v in water), 5 mL; Orange G (1% w/v in water), 0.5 mL; phenol, 5 g; chloral hydrate, 5 g; glacial acetic acid, 2 mL; glycerol, 25 mL; distilled water, 50 mL) were added. Stained pollen grains were observed with a Zeiss Axio Imager A1 microscope equipped with differential contrast interference microscopy optics. After staining viable pollen is purple, while dead pollen is green.

### Embryo Development

For embryo development, siliques at variable developmental stages were removed from Col-0, *fra2, lue1* and *ktn1-2* plants, fixed in 50% v/v ethanol and 10% v/v acetic acid in water and cleared in chloral hydrate solution as described above. Following clearing, siliques were dissected in a drop of chloral hydrate solution on a glass slide to extract ovules. Samples were imaged and documented with DIC optics of a Zeiss AxioImager microscope equipped with a Zeiss MRc5 digital camera.

### Cloning of pKTN1::GFP:KTN1 Construct

The N-terminal fusion construct of *enhanced GFP (eGFP)* with *AtKTN1* driven under its own promoter (*pKTN1::GFP:KTN1*) was prepared using genomic DNA from leaf tissue. The 1143 bp *AtKTN1* (AT1G80350) promoter upstream of ATG start codon was amplified using respective primers: 5′-GGGGACAACTTTGTATAGAAAAGTTGTGCCTGCAGATAGCTTACTCAG-3′ and 5′-GGGGACTGCTTTTTTGTACAAACTTGGCCTCTTTTACTAAAAAAATAGCC-3′.

AtKTN1 genomic sequence for N-terminal fusion (*GFP* is fused with N-terminus of *KTN1*) was amplified using primers:5′-GGGGACAGCTTTCTTGTACAAAGTGGGCATGGTGGGAAGTAGTAATTCGTTAGCC-3′ and 5′-GGGGACAACTTTGTATAATAAAGTTGCTTAAGCAGATCCAAACTCAGAGAG-3′.

Amplified promoter, *GFP* sequence (plasmid pEN-L1-F-L2 MultiSiteGateway^®^) and *AtKTN1* genomic DNA were assembled using recombination reaction according to MultiSite Gateway^®^ Three-Fragment Vector Construction Kit and cloned into pB7m34GW.0, which was then used for *Agrobacterium tumefaciens* GW3101 transformation. Col-0 and *KATANIN 1* mutants were transformed with this construct using established floral dip method.

## Results

### Abberant Fertility of KATANIN 1 Mutants

**FIGURE 1:**
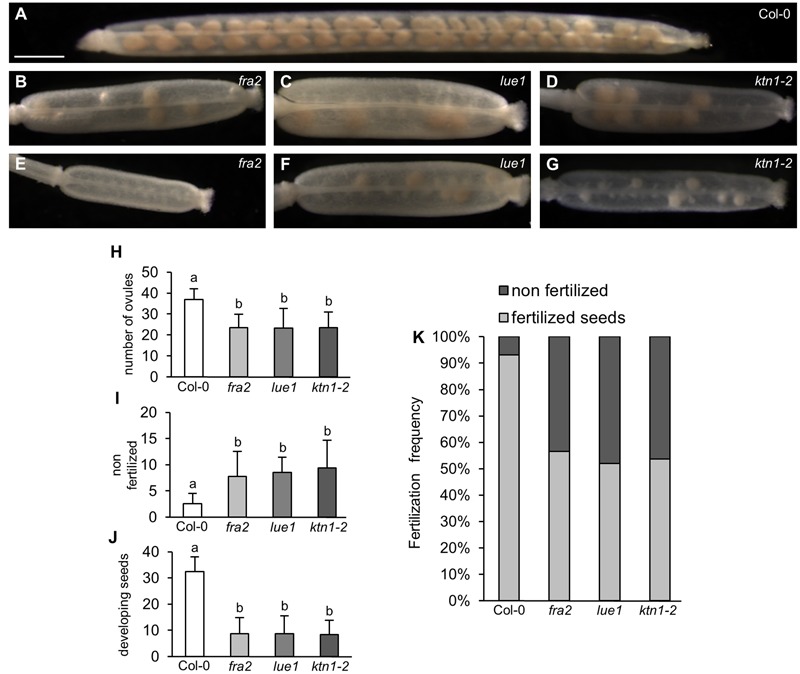
**Development of siliques and fertilized seeds in *KATANIN 1* mutants. (A–G)** Representative images of developing seeds in cleared siliques of Col-0 **(A)** and *KATANIN 1* mutants: *fra2* **(B)**, *lue1* **(C)**, and *ktn1-2* **(D)**, and asynchronous seed development in *fra2* **(E)**, *lue1* **(F)**, and *ktn1-2* **(G)**. **(H)** Number of total ovules per silique in Col-0 and *KATANIN 1* mutants. **(I)** Number of non-fertilized seeds per silique in Col-0 and *KATANIN 1* mutants. **(J)** Number of developing seeds per silique in Col-0 and *KATANIN 1* mutants. **(K)** The ratio of non-fertilized and fertilized seeds in siliques of Col-0 and *KATANIN 1* mutants. Final calculations were based on data collection from 13 to 29 siliques. Different lowercase letters indicate statistical significance between treatments (*p* < 0.001). Error bars show ± SD. Scale bar = 1 mm.

All *KATANIN 1* mutants showed reduced fertility, which is evident by the formation of numerous non-elongating siliques on the inflorescences of *fra2, lue1* and *ktn1-2*, suggesting that irregular development of siliques arised from defects in fertilization (Supplemental Figures 1A–D). In all three mutants, *fra2, lue1* and *ktn1-2*, mature siliques were shorter compared to Col-0 (**Figures 1A–G** and Supplemental Figures 1A–H). Col-0 siliques showed synchronous seed development (**Figure 1A**), but in *fra2, lue1* and *ktn1-2* the number of ovules per silique was roughly half (**Figures 1A–H**). More detailed studies showed unfertilized ovules (**Figures 1I,K**) containing only few developing seeds (**Figures 1B–G,J,K**).

### Defects in Ovule Development in KATANIN 1 Mutants

**FIGURE 2:**
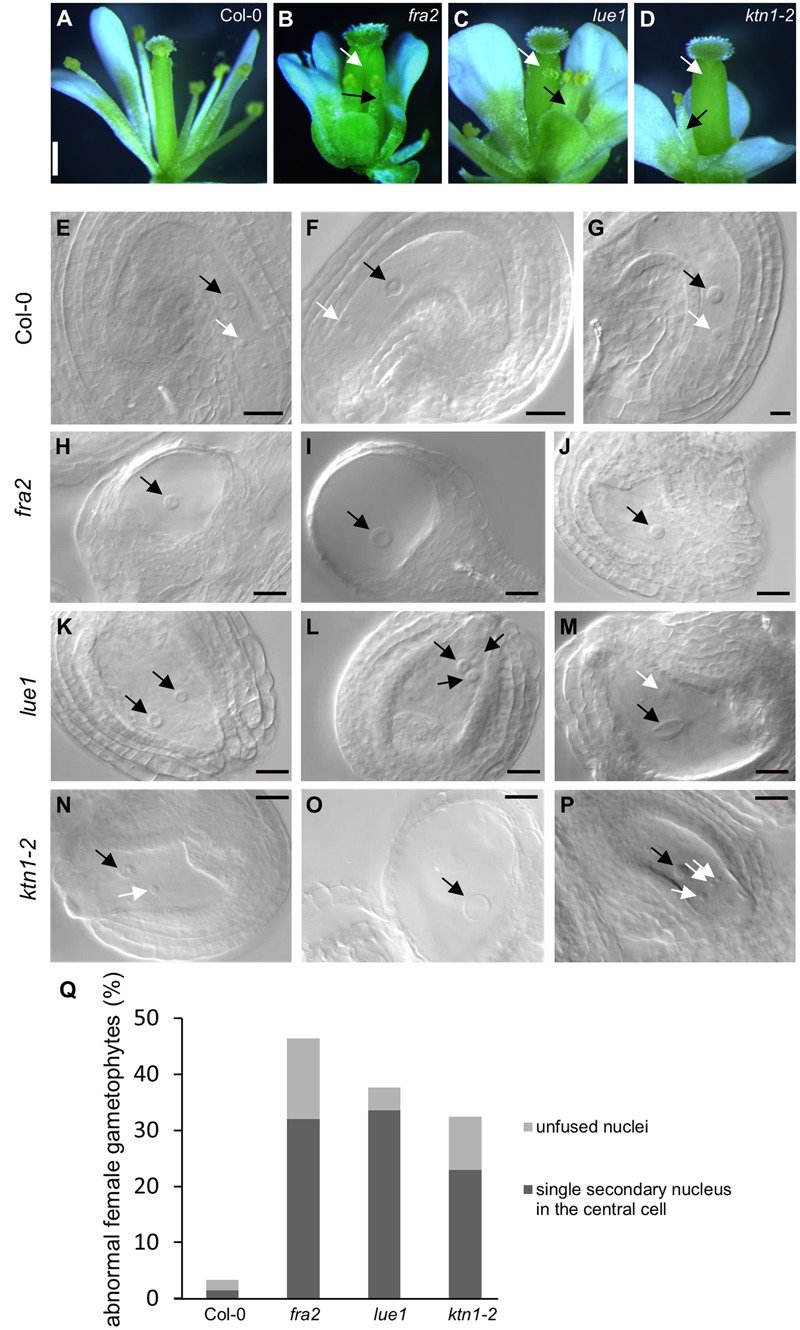
**Developmental defects in flowers and embryo sacs of *KATANIN 1* mutants. (A–D)** Representative pictures images of flowers of Col-0 **(A)** and *KATANIN 1* mutants *fra2* **(B)**, *lue1* **(C)**, and *ktn1-2* **(D)**. Defective stamens and pistils are depicted with black and white arrows. **(E–G)** Wild type embryo sacs. The central cell nucleus (black arrows) and the egg cell nucleus (white arrows) were in proximal distance close to the micropylar pole showing that the migration and the precise positioning of the polar nuclei have been normally performed. **(H–J)** *fra2* embryo sacs showed abnormalities in the migration and the positioning of nuclei and in the most cases only the central cell nucleus was present. Abnormalities in the development of the outer and inner integuments have been also observed **(H,I)**. **(K–M)** *lue1* embryo sacs. Misplacement of the polar nuclei **(K)** and unfused or double central cell nuclei **(L,M)**. **(N–P)** *ktn1-2* embryo sacs. Misplacement of the polar nuclei **(N)**, unfused nuclei **(P)**, highly vacuolated central cell nuclei, absence of egg cell nucleus **(O)**, abnormalities in the development of the outer and inner integuments **(N–P)**. **(Q)** Quantification of the abnormal ovule phenotypes. Scale bars = 500 μm **(A–D)**; 50 μm **(E–P)**.

In mature flowers of *KATANIN 1* mutants the pistils display developmental defects, such as reduced size and abnormal carpel junction (**Figures 2B–D**), while the stamens grow separately from the female organs showing decreased filament length unable to reach the pistils for successful pollination (**Figures 2A–D**), as previously described (Qu et al., 2012). There were some differences among the *KATANIN 1* mutants in the flower patterning and the development of the four whorls constituting flower, however, the overall picture of developed flowers was generally compromised. To gain insight into the low fertility defects in depth analysis of female and male reproductive tissues was conducted. Thorough phenotypic characterization of unpollinated ovule formation in *KATANIN 1* mutants compared to Col-0 (**Figures 2E–Q**) revealed that ovule development was variably defective in *KATANIN 1* mutants, ranging from severely malformed to normal and fertile. In detail female reproductive tissue analysis showed the following: 32% of the unfertilized ovules in *fra2* showed a single secondary nucleus in their central cells and 14.4% showed unfused nuclei (**Figures 2H–J,Q**), in *lue1* ovules, 33.6% contained a single secondary nucleus in their central cells and 4% contained unfused nuclei (**Figures 2K–M,Q**), while in *ktn1-2* the percentages were 22.97 and 9.45%, respectively (**Figures 2N–P,Q**). Wild type plants showed very low occurence of such malformed ovules, 1.5 and 1.8%, respectively (**Figure 2Q**). Together, these observations indicate problems with migration and the precise positioning of nuclei and probably in the fusion of the polar nuclei. Such malfunctions can create problems in the attraction and guidance of the sperm cells within the embryo sac. Moreover, analysis of *KATANIN 1* mutant ovules also revealed deformed inner and outer integuments in *fra2, lue1*, and *ktn1-2* mutants suggesting sporophytic defects in the development of embryo sac (Supplemental Figures 2A–D).

### Anther Development in KATANIN 1 Mutants

**FIGURE 3:**
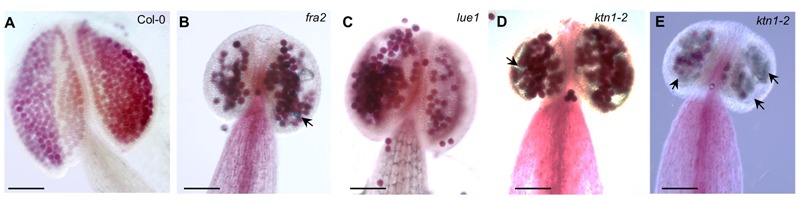
**Developmental defects in anthers of *KATANIN 1* mutants. (A–E)** Representative images of Alexander solution stained pollen in anthers of Col-0 **(A)** and *KATANIN 1* mutants *fra2* **(B)**, *lue1* **(C)**, and *ktn1-2* **(D,E)**. Arrows point to the unviable shrunken pollen stained green by Alexander solution **(B,D,E)**. Scale bars = 100 μm **(A–E)**.

Since KATANIN p80 is essential for male fertility and sperm production in mouse (O’Donnell et al., 2012), we checked the formation of viable pollen in mature anthers of *fra2, lue1* and *ktn1-2* mutants. Developed mature anther on the short stamens with stunt wider filaments of *KATANIN 1* mutants, displayed also abnormal growth, as anther lobes were non-symmetrically and irregularly developed (**Figures 3A–E**). Alexander staining assays showed variable pollen viability in mature anther lobes of *fra2, lue1* and *ktn1-2* as compared to the wild type, while degenerated inviable pollen could be also observed (**Figures 3B,D,E**).

### Aberrant Embryo Development in KATANIN 1 Mutants

**FIGURE 4:**
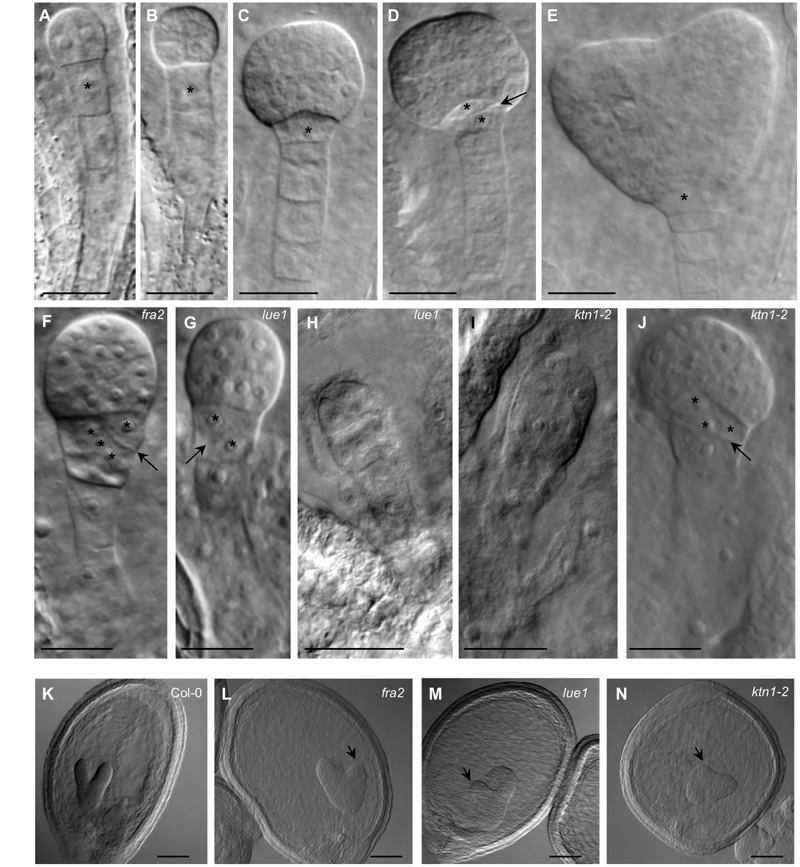
**Embryonic developmental defects in *KATANIN 1* mutants. (A–E)** Embryos of Col-0 at different developmental stages including 2 to 4-cells **(A)**, octant **(B)**, early globular **(C)**, transition **(D)**, and heart shaped **(E)**, showing typical organization of embryo proper and suspensor. Asterisks mark the position of the first suspensor cell **(A,B)** and the hypophysis in later stages **(C–E)**. Transversal cell division of the hypophysis giving rise to quiescent center and columella stem cell is indicated by arrow **(D)**. **(F)** Early globular embryo of the *fra2* mutant showing slightly abnormal embryo proper and oblique cell division (arrow) at the hypophysis (asterisks). **(G)** Embryo of the *lue1* mutant at early globular stage showing oblique cell division (arrow) at the hypophysis (asterisks). **(H)** Abnormaly elongated preglobular embryo of the *lue1* mutant. **(I)** Abnormaly elongated preglobular embryo of the *ktn1-2* mutant. **(J)** Globular embryo of the *ktn1-2* mutant showing oblique cell division (arrow) at the hypophysis (asterisks). **(K–N)** The embryos at the heart shape stage of wild type Col-0 **(K)** and *KATANIN 1* mutants *fra2* **(L)**, *lue1* **(M)** and *ktn1-2* **(N)**. Note the asymmetry of the globular embryos in *KATANIN 1* mutants (arrows). Scale bars = 20 μm.

Embryonic development in *KATANIN 1* mutants and in Col-0 was examined with DIC optics. In Col-0 it was possible to discern several stages of embryo development which progressed orderly (ten Hove et al., 2015; **Figures 4A–E**). In all *KATANIN 1* mutants we documented abnormalities in the embryo development, like misoriented cell divisions in the proembryo and the hypophysis (**Figures 4F–J**) and abnormally shaped hypophysis (**Figures 4F,G,J**). In severe cases, particularly in the *ktn1-2* mutant (**Figures 4I,J**), the shape of the embryo proper significantly deviated from its typical spherical form (**Figure 4C**). The redundant cell divisions in the ealry stages of embryogenenesis lead to malformed embryos of later developmental stages like heart shape stage (**Figures 4L–N**) compared to Col-0 (**Figure 4K**).

### Abberant Seed Formation in KATANIN 1 Mutants

**FIGURE 5:**
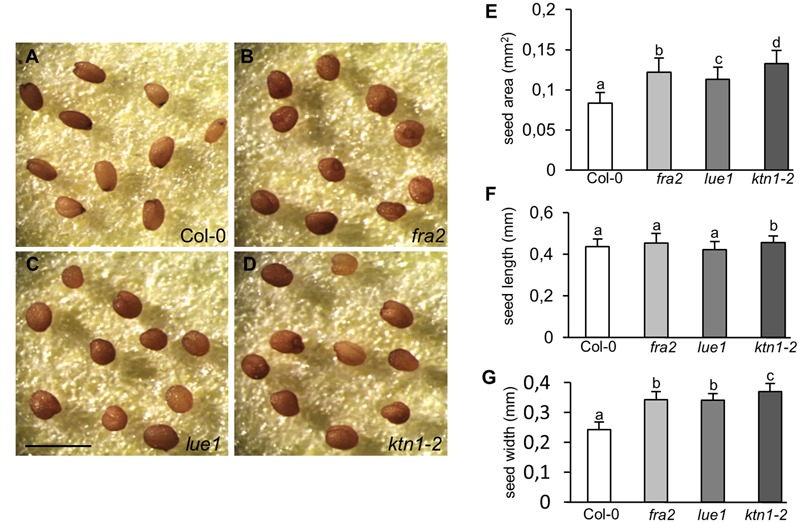
**Abnormal seed development in *KATANIN 1* mutants. (A–D)** Representative images of mature seeds in Col-0 and *KATANIN 1* mutants. **(A)** Seeds of Col-0 exhibited a smooth contour and an elongated shape. **(B–D)** Seeds of *fra2* **(B)**, *lue1* **(C)**, and *ktn1-2* **(D)** appeared more rounded and anomalous. **(E)** Seed area in Col-0 and *KATANIN 1* mutants. **(F)** Seed length in Col-0 and *KATANIN 1* mutants. **(G)** Seed width in Col-0 and *KATANIN 1* mutants. Final calculations were based on data collection from 37 to 57 seeds. Different lowercase letters indicate statistical significance between treatments (*p* < 0.05). Error bars show ± SD. Scale bar = 1 mm.

Consistently with the previously described defects, the development of viable seeds is considerably compromised in *KATANIN 1* mutants compared to Col-0. Mature seeds of *fra2, lue1* and *ktn1-2* were misshaped compared to Col-0 (**Figures 5A–D**), showing rounding and anomalous contour. Seed size analysis showed that *KATANIN 1* mutants produce larger seeds compared to wild type. Quantification of the seed size parameters showed that all *KATANIN 1* mutants display significantly larger seed areas (**Figures 5A–E** and Supplementary Table S1). In comparison to Col-0, seed length and width are differentially affected in *KATANIN 1* mutants with the seed width to exhibit the most striking increase (**Figures 5A–E**). Differences in seed form parameters could be also observed among *fra2, lue1*, and *ktn1-2* (**Figures 5E–G**).

### Genetic Complementation and Rescue of Mutant Phenotypes

**FIGURE 6:**
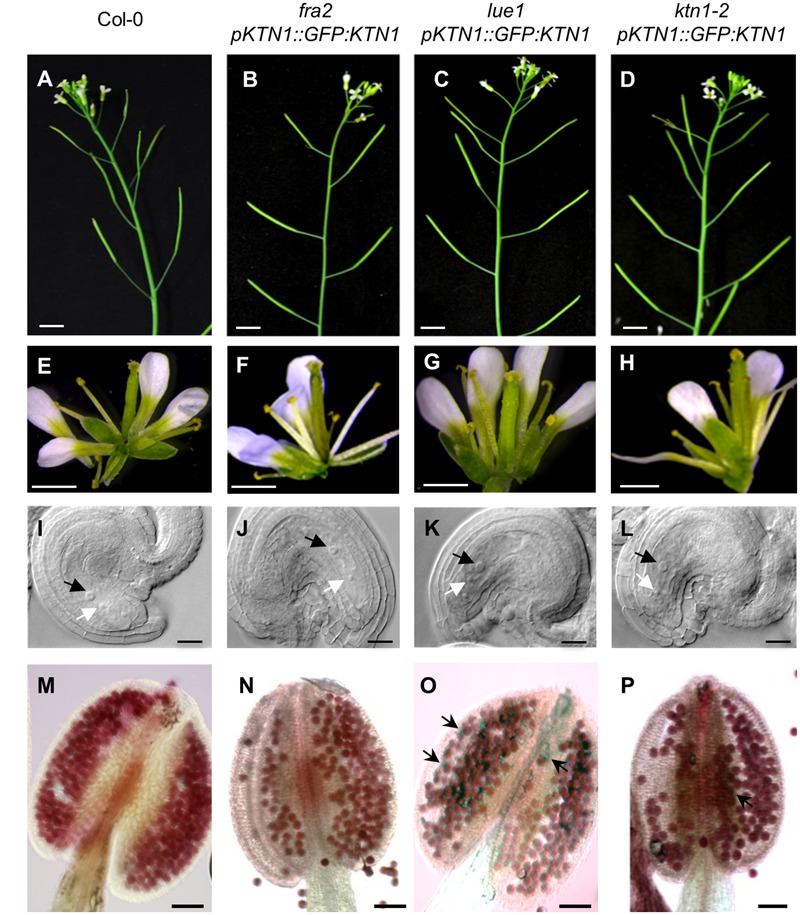
**Genetic complemetation of silique, flower, embryo sac and anther developmental defects in *KATANIN 1* mutants using *pKTN1::GFP:KTN1* construct. (A–D)** Influorescence in wild-type (Col-0) plants **(A)** and rescued *fra2* **(B)**, *lue1* **(C)**, and *ktn1-2* **(D)** mutants. **(E–H)** Flowers in wild-type (Col-0) plants **(E)** and rescued *fra2* **(F)**, *lue1* **(G)** and *ktn1-2* **(H)** mutants. **(I–L)** Ovules in wild-type (Col-0) plants **(I)** and geneticaly complemented *fra2* **(J)**, *lue1* **(K)** and *ktn1-2* **(L)** mutants. Black and white arrows point to the central cell and egg cell, respectively **(I–L)**. **(M–P)** Anthers in wild-type (Col-0) plants **(M)** and rescued *fra2* **(N)**, *lue1* **(O)** and *ktn1-2* **(P)** mutants. Black arrows point to the unviable pollen grains **(O,P)**. Scale bars = 5 mm **(A–D)**, 1 mm **(E–H)**, 50 μm **(I–L)**, 100 μm **(M–P)**.

To test whether GFP-KTN1 fusion protein can complement described phenotypes of *KATANIN 1* mutants we transformed *fra2, lue1* and *ktn1-2* plants with *pKTN1::GFP:KTN1* construct. We analyzed T2 generation of transformed *fra2, lue1* and *ktn1-2* mutants carrying *pKTN1::GFP:KTN1* constructs and we selected for further phenotypic analyses plants that harbor a single T-DNA copy (*n* > 200, Chi-squared test *p* < 0.05). The phenotypes of *fra2, lue1* and *ktn1-2* mutants could be rescued by introducing *pKTN1::GFP:KTN1* construct. In detail, adult plants showed a wild type inflorescence architecture (**Figures 6A–D**) and flower development (**Figures 6E–H**). Concerning development of reproductive organs, introduction of *pKTN1::GFP:KTN1* rescued malformed phenotypes of ovules and anthers in *KATANIN 1* mutants described above (**Figures 6I–P**). Nevertheless, in *lue1* and *ktn1-2* genetically complemented plants harboring *pKTN1::GFP:KTN1* one can still detect some inviable pollen by Alexander staining, though in very low levels (**Figures 6O,P**).

**FIGURE 7:**
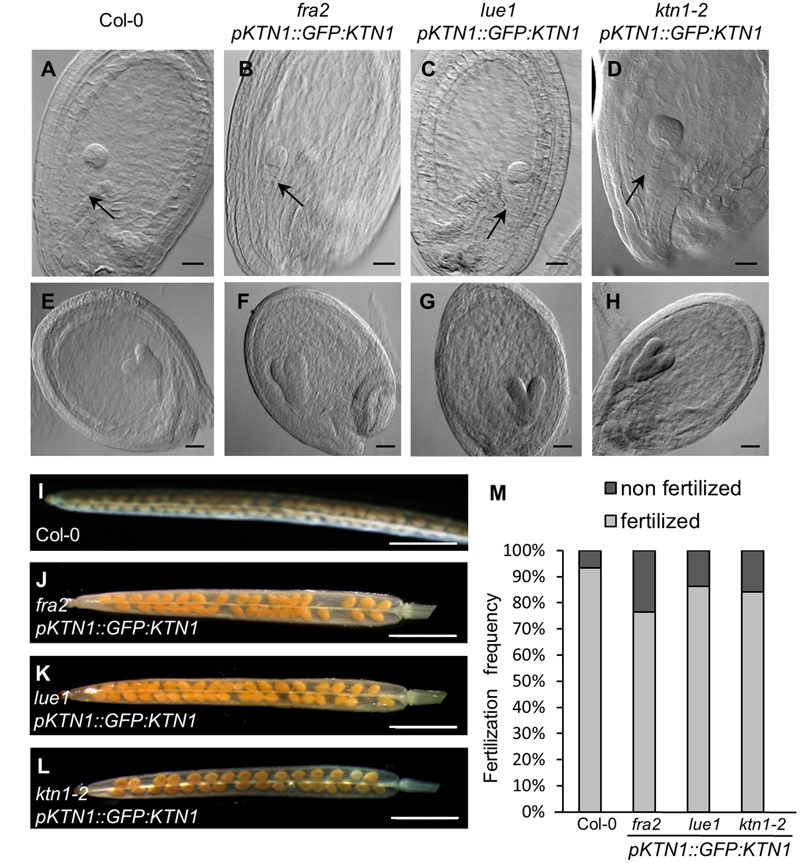
**Genetic complementation of embryo and seed developmental defects in *KATANIN 1* mutants using *pKTN1::GFP:KTN1* construct. (A–D)** Globular stage of embryo development in wild-type (Col-0) plants **(A)** and rescued *fra2* **(B)**, *lue1* **(C)**, and *ktn1-2* **(D)** mutants. Black arrows point to the suspenzor **(A–D)**. **(E–H)** Globular stage of embryo development in wild-type (Col-0) plants **(E)** and rescued *fra2* **(F)**, *lue1* **(G)**, and *ktn1-2* **(H)** mutants. **(I–L)** Siliques in wild-type (Col-0) plants **(I)** and rescued *fra2* **(J)**, *lue1* **(K)** and *ktn1-2* **(L)** mutants. **(M)** Fertilization frequency in wild-type (Col-0) plants **(I)** and rescued *fra2* **(J)**, *lue1* **(K),** and *ktn1-2* **(L)** mutants. Scale bars = 20 μm **(A–H)**, 1 mm **(I–L)**.

Next, we checked development of seeds in such complemented *fra2, lue1* and *ktn1-2* mutants and we observed rescue of embryo development (**Figures 7A–H**). Additionally, the complemented *KATANIN 1* mutants displayed increased levels of fertilized seeds comparable to the wild type (**Figures 7I–M**) with minor differences among the mutants (**Figure 7M**).

**FIGURE 8:**
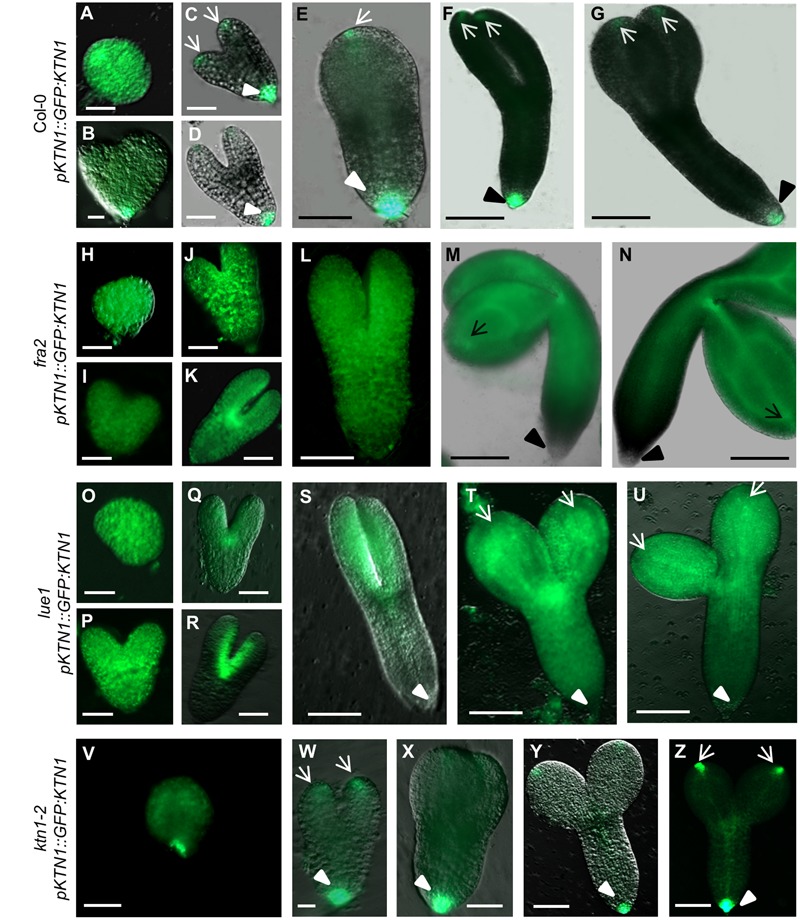
***KATANIN 1* expression in embryos in wild type and genetically complemented *KATANIN 1* mutants. (A–F)** different embryo developmental stages in wild-type (Col-0) plants expressing *pKTN1::GFP:KTN1* construct: globular stage **(A)**, triangle stage **(B)**, heart stage **(C)**, early torpedo **(D)**, late torpedo **(E)**, premature **(F)** and mature embryos **(G)**. **(H–N)** different embryo developmental stages in rescued *fra2* mutant plants expressing *pKTN1::GFP:KTN1* construct: globular stage **(H)**, triangle stage **(I)**, heart stage **(J)**, early torpedo **(K)**, late torpedo **(L)**, premature **(M)**, and mature embryos **(N)**. **(O–U)** GFP-KTN1 fusion protein localization in different embryo developmental stages in rescued *lue1* mutant plants expressing *pKTN1::GFP:KTN1* construct: globular stage **(O)**, triangle stage **(P)**, heart stage **(Q)**, early torpedo **(R)**, late torpedo **(S)**, premature **(T)**, and mature embryos **(U)**. **(V–Z)** different embryo developmental stages in rescued *ktn1-2* mutant plants expressing *pKTN1::GFP:KTN1* construct: globular stage **(V)**, heart stage **(W)**, torpedo **(X)**, premature **(Y)**, and mature embryos **(Z)**. Arrows indicate cotyledon poles, arrow-heads indicate embryonic root meristem **(A–Z)**. Scale bars = 20 μm **(A–D, H–K, O–R, V–W)** and 100 μm **(E–G, L–N, S–U, X–Z)**.

**FIGURE 9:**
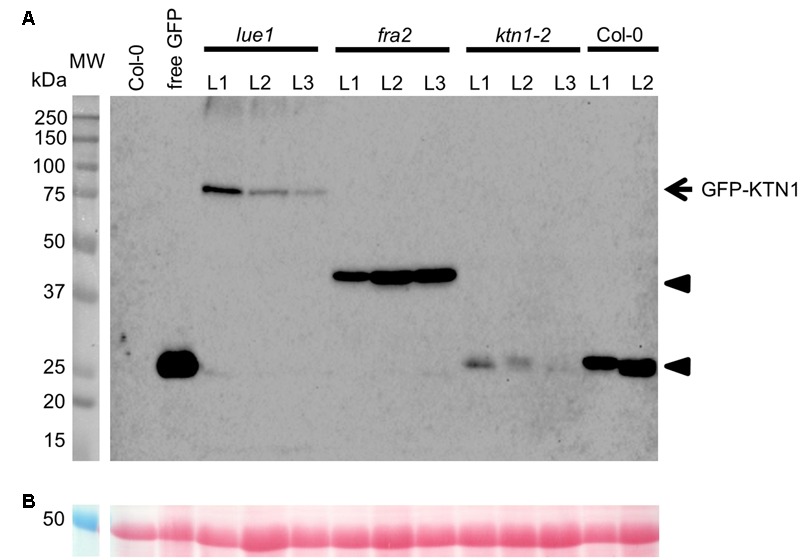
**Western blot detection of GFP-KTN1 in genetically complemented *fra2, lue1*, and *ktn1-2* mutants carrying *pKTN1::GFP:KTN1* construct. (A)** GFP-KTN1 protein (85 kDa) confirmed by using anti-GFP antibody (arrow) shows the chimeric GFP-KTN1 protein in *lue1* genetic background while arrow heads show truncated and cleaved forms of GFP-KTN1 in *fra2, ktn1-2* and Col-0. **(B)** Loading control (proteins stained by Ponceau).

Analysis of KATANIN 1 expression profile in the developing embryos of wild type (Col-0) transformed with *pKTN1::GFP:KTN1* showed that it is expressed in all embryonic cells during the globular stage (**Figure 8A**), at the triangle stage there is induction of KATANIN 1 expression at the embryonic root (**Figure 8B**) and in later developmental stages (heart to mature embryos) we could detect polarized KATANIN 1 expression in cotyledon poles and embryonic root (**Figures 8C–G**). These findings can help to explain the aberrant phenotypes of developing embryos in *KATANIN 1* mutants. KATANIN 1 expression pattern analysis in genetically complemented mutants showed that in *ktn1-2* (**Figures 8V–Z**) it was consistent with polar patterning in wild type (**Figures 8A–G**). In the case of genetically complemented *fra2* and *lue1* KATANIN 1 expression differed as it was generally distributed in most parts of the embryo while it was more prominent in the shoot apical meristem but almost not detectable in the embryonic root tip (**Figures 8H–U**). Western blot analysis for the detetion of GFP-KTN1 chimeric protein in the complemented *fra2, lue1* and *ktn1-2* verified the presence of full-length GFP-KTN1 fusion protein only in *lue1* genetic background, while in *fra2, ktn1-2* and Col-0 we were able to detect only truncated forms of the GFP-KTN1 protein (in the case of *fra2*) or likely GFP alone driven by *KATANIN 1* promoter (**Figure 9**). These findings suggest some complex unknown mechanisms involved in the regulation of expression pattern and distribution of KATANIN 1 protein in such plant lines.

## Discussion

Although the role of KATANIN 1 in the male and female gametogenesis is emerging in animal systems (O’Donnell et al., 2012; Joly et al., 2016; Tang et al., 2016) such function has not yet been found in plants. Future detailed genetic studies might eventually shed more light on gametophytic defects in the *KATANIN 1* mutants. In this study, however, we focus on sporophytic developmental phenotypes of *KATANIN 1* mutants during embryogenesis and seed formation including a thorough description of developmental defects which are genetically rescued by appropriate *pKTN1::GFP:KTN1* functional construct.

### KATANIN 1 Role in Embryo Sac Polarity

The regulation of microtubule organization and dynamics is critical for many cellular processes during plant development. Gametogenesis is the process by which gametes undergo mitotic and meiotic division and differentiation to produce germ units capable of motility and fertilization. Studies have shown that the microtubule-based cytoskeleton that provides the track for the transport of spermatids and cell organelles during spermatogenesis is likely working in concert with the actin cytoskeleton (Tang et al., 2013, 2016; O’Donnell and O’Bryan, 2014). The microtubule cytoskeleton is essential for polar organelle distribution including arrangement and migration of nuclei in developing embryo sac (Russell, 1993; Webb and Gunning, 1994). Moreover, radial perinuclear microtubules support cellularization that leads to the formation of the seven-celled embryo sac (Webb and Gunning, 1994). Extensive arrays of microtubules are present throughout the megagametogenesis and can be categorized into the cytoplasmic microtubules and the nuclear-associated networks typical for egg cell and two- and four-nucleate stages (Webb and Gunning, 1994). They possibly regulate the migration and precise positioning of nuclei in these multinucleate cells and the sperm cell guidance within the degenerated synergid (Webb and Gunning, 1994). So, based on cytological observations the defects detected in *KATANIN 1* mutant embryo sacs can be attributed to the lack of rapid microtubule rearranging ability due to the missing microtubule severing activity.

Moreover, we show here that these embryo sac polarity defects were resued by *pKTN1::GFP:KTN1* construct.

### KATANIN 1 Role in Embryo and Seed Development

Cytoskeletal rearrangements and dynamic changes have been rarely studied during diverse stages of plant fertilization and embryo development. Fertilization triggers the completion of meiosis while short acentrosomal meiotic spindle must be disassembled and right afterward replaced by big mitotic spindle filling most of the zygote volume (Albertson, 1984; Kemphues et al., 1986; Albertson and Thomson, 1993; McCarter et al., 1999; Yang et al., 2003). This quick microtubule remodeling has to be under precise developmental control.

In animal systems recent studies have shed light into the role of katanin severing complex during the female meiotic spindle assemby (Joly et al., 2016) in *Caenorhabditis elegans* and during sperm production in mouse (O’Donnell et al., 2012). Additionally, katanin complex severing microtubules is under tight regulation during the transition form the meiotic to mitotic stage to allow proper embryogenesis, as its persistence could have detrimental effects (Beard et al., 2016). Previous studies in Arabidopsis roots showed that *fra2* and *lue1* mutants show mitotic spindle multipolarity and rotation compared to Col-0, suggesting that aberrant spindle positioning in *KATANIN 1* mutants may be the mechanistic basis for misorientation of the cell division plane (Panteris et al., 2011; Panteris and Adamakis, 2012). The strict pattern of cell divisions guiding development of wild-type embryos has been well previously described (Jürgens and Mayer, 1994). In this study we report that *KATANIN 1* mutants show defects in cell division plane orientation not restricted to particular stages or cells of the developing embryos, resulting in malformation of embryos. Concomitantly seed development is also compromised in *KATANIN 1* mutants.

Frequencies and patterns of cell divisions in embryo, endosperm and integuments dictate size of seeds. It has also been reported that ovule abortion or embryo lethality is correlated with the production of enlarged seeds (Sornay et al., 2016). Given the problems described in *KATANIN 1* mutant with ovule abortion and embryo development the formation of enlarged seeds corroborates these results.

Importantly, most of above-described abberant embryo and seed phenotypes in *KATANIN 1* mutants were almost fully (in the case of *lue1* possessing full-length GFP-KTN1) or at least partialy (in the case of *fra2* and *ktn1-2*) genetically rescued by functional *pKTN1::GFP:KTN1* construct. Although GFP-KTN1 chimeric protein might be cleaved (in the case of *ktn1-2*) or truncated (in the case of *fra2*) it can still complement mutant phenotypes. Moreover, results from developing embryos might suggest that patterning and spatiotemporal distribution of GFP driven by *KATANIN 1* promoter might differ from those of full-length or truncated KATANIN 1 proteins driven by the same promoter.

## Author Contributions

IL carried out most of the experiments conducted herein. GK helped with embryo preparations and with microscopy in general. JŠ conceived the project and supervised its progress. DS and JŠ wrote the manuscript with input by the other authors.

## Conflict of Interest Statement

The authors declare that the research was conducted in the absence of any commercial or financial relationships that could be construed as a potential conflict of interest.
