# Supplementary material for: KATANIN 1 Is Essential for Embryogenesis and Seed Formation in Arabidopsis
Source: Front Plant Sci. 2017 May 5;8:728. doi: 10.3389/fpls.2017.00728 (PMC5418335; doi:10.3389/fpls.2017.00728)
Supplement: Supplementary file 1 [file Table_1.DOCX]

**Supplementary Table**

**Table S1.** Seed size parameters of Col-0 and *KATANIN 1* mutants.

| Seed | Col-0 | | *fra2* | | *lue1* | | *ktn1-2* | |
| --- | --- | --- | --- | --- | --- | --- | --- | --- |
| area (mm^2^) | 0,083 | ±0,014 | 0,122 | ±0,018 | 0,113 | ±0,015 | 0,132 | ±0,016 |
| length (mm) | 0,436 | ±0,036 | 0,453 | ±0,048 | 0,422 | ±0,039 | 0,456 | ±0,033 |
| width (mm) | 0,242 | ±0,025 | 0,342 | ±0,027 | 0,342 | ±0,021 | 0,369 | ±0,028 |
