# Supplementary material for: KATANIN 1 Is Essential for Embryogenesis and Seed Formation in Arabidopsis
Source: Front Plant Sci. 2017 May 5;8:728. doi: 10.3389/fpls.2017.00728 (PMC5418335; doi:10.3389/fpls.2017.00728)
Supplement: Supplementary file 2 [file Presentation_1.pdf]

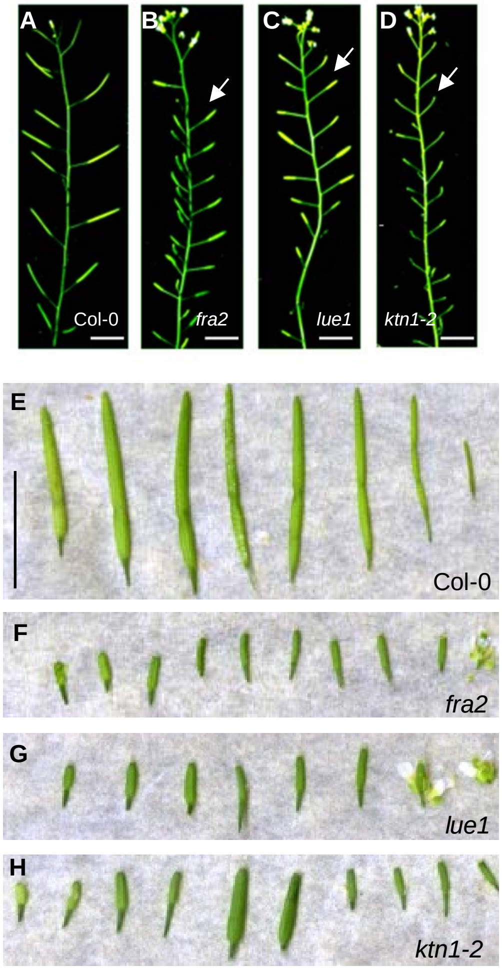

Supplemental Figure 1. Phenotype of siliques in Col-0 and *KATANIN 1* mutants.
(A-D) Representative pictures of inflorescences of Col-0 **(A)** and *KATANIN 1* mutants, *fra2* **(B)**, *lue1* **(C)** and *ktn1-2* **(D)**. Note short siliques in *fra2*, *lue1*, and *ktn1-2* mutants (white arrows). **(E-H)** Representative pictures of all siliques (youngest on the right and oldest on the left) from the 2nd branch of the stem of Col-0 and *KATANIN 1* mutants from 6 week old plants. **(E)** Siliques of Col-0 exhibited normal size according their developmental order and respective position on the stem. **(F-H)** Siliques of *fra2* **(F)**, *lue1* **(G)** and *ktn1-2* **(H)** were significantly shorter. Scale bars = 1 cm.

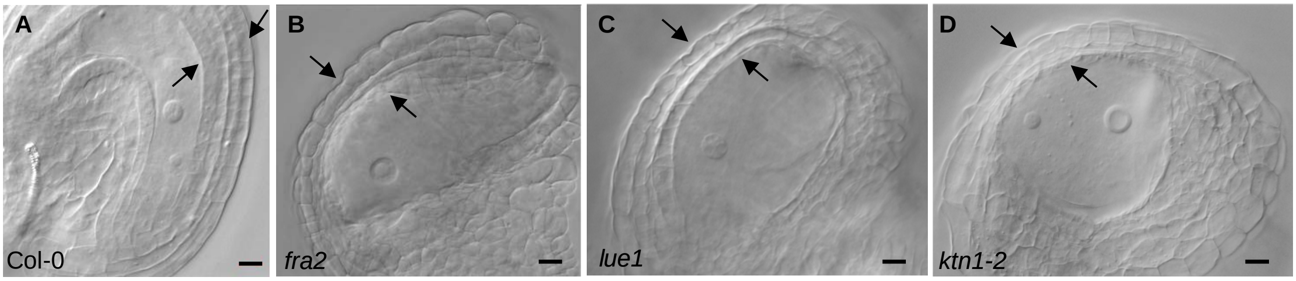

Supplemental Figure 2. Ovule integument defects in *KATANIN 1* mutants.
(A-D) Representative pictures of ovules of Col-0 **(A)** and *KATANIN 1* mutants *fra2* **(B)**, *lue1* **(C)** and *ktn1-2* **(D)**. Arrows point to the deformed inner and outer integuments. Scale bars = 20 µm.
